# Involvement of Capsaicin-Sensitive Lung Vagal Neurons and TRPA1 Receptors in Airway Hypersensitivity Induced by 1,3-β-D-Glucan in Anesthetized Rats

**DOI:** 10.3390/ijms21186845

**Published:** 2020-09-18

**Authors:** You Shuei Lin, I-Hsuan Huang, Sheng-Hsuan Lan, Chia-Ling Chen, Yueh-Yin Chen, Nai-Ju Chan, Chun-Chun Hsu

**Affiliations:** 1 Department of Physiology, School of Medicine, College of Medicine, Taipei Medical University, Taipei 110, Taiwan; yslin@tmu.edu.tw; 2 Graduate Institute of Medical Sciences, College of Medicine, Taipei Medical University, Taipei 110, Taiwan; d119108007@tmu.edu.tw (Y.-Y.C.); d119108008@tmu.edu.tw (N.-J.C.); 3 School of Respiratory Therapy, College of Medicine, Taipei Medical University, Taipei 110, Taiwan; b117107009@tmu.edu.tw (I.-H.H.); b117107014@tmu.edu.tw (S.-H.L.); chialing66@tmu.edu.tw (C.-L.C.); 4 Division of Pulmonary Medicine, Department of Internal Medicine, Taipei Medical University Hospital, Taipei 110, Taiwan

**Keywords:** airway hypersensitivity, capsaicin-sensitive lung vagal afferents, afferent sensitization, fungi, glucan, sensory neuron, TRPA1, Dectin-1

## Abstract

Airway exposure to 1,3-β-D-glucan (β-glucan), an essential component of the cell wall of several pathogenic fungi, causes various adverse responses, such as pulmonary inflammation and airway hypersensitivity. The former response has been intensively investigated; however, the mechanism underlying β-glucan-induced airway hypersensitivity is unknown. Capsaicin-sensitive lung vagal (CSLV) afferents are very chemosensitive and stimulated by various insults to the lungs. Activation of CSLV afferents triggers several airway reflexes, such as cough. Furthermore, the sensitization of these afferents is known to contribute to the airway hypersensitivity during pulmonary inflammation. This study was carried out to determine whether β-glucan induces airway hypersensitivity and the role of the CSLV neurons in this hypersensitivity. Our results showed that the intratracheal instillation of β-glucan caused not only a distinctly irregular pattern in baseline breathing, but also induced a marked enhancement in the pulmonary chemoreflex responses to capsaicin in anesthetized, spontaneously breathing rats. The potentiating effect of β-glucan was found 45 min later and persisted at 90 min. However, β-glucan no longer caused the irregular baseline breathing and the potentiating of pulmonary chemoreflex responses after treatment with perineural capsaicin treatment that blocked the conduction of CSLV fibers. Besides, the potentiating effect of β-glucan on pulmonary chemoreflex responses was significantly attenuated by N-acetyl-L-cysteine (a ROS scavenger), HC-030031 (a TRPA1 antagonist), and Laminarin (a Dectin-1 antagonist). A combination of Laminarin and HC-030031 further reduced the β-glucan-induced effect. Indeed, our fiber activity results showed that the baseline fiber activity and the sensitivity of CSLV afferents were markedly elevated by β-glucan instillation, with a similar timeframe in anesthetized, artificially ventilated rats. Moreover, this effect was reduced by treatment with HC-030031. In isolated rat CSLV neurons, the β-glucan perfusion caused a similar pattern of potentiating effects on capsaicin-induced Ca^2+^ transients, and β-glucan-induced sensitization was abolished by Laminarin pretreatment. Furthermore, the immunofluorescence results showed that there was a co-localization of TRPV1 and Dectin-1 expression in the DiI-labeled lung vagal neurons. These results suggest that CSLV afferents play a vital role in the airway hypersensitivity elicited by airway exposure to β-glucan. The TRPA1 and Dectin-1 receptors appear to be primarily responsible for generating β-glucan-induced airway hypersensitivity.

## 1. Introduction

Airway exposure to fungi is one of the major causes of airway hypersensitivity [1,2,3,4,5], a key syndrome during airway inflammation [6,7,8]. However, the underlying mechanism of the fungal-induced airway hypersensitivity is still unclear. 1, 3-β-D-glucan (β-glucan) is the most abundant polysaccharide originating from the cell walls of fungi [9]. By activation of Dectin-1 receptors, β-glucan is mainly responsible for triggering fungi-induced airway inflammation [10,11]. Dectin-1 was identified and reported by Brown and Gordon as a glucan receptor [10,12]. It has been reported that airway exposure to β-glucan leads to lung damage and lung inflammation [5,13,14]. Besides, the β-glucan level is significantly elevated in patients with adverse pulmonary symptoms, including lung inflammation and airway hyperresponsiveness [15,16]. Therefore, it is believed that β-glucan acts as a potent pro-inflammatory inducer, which can induce the production of several inflammatory cytokines, such as reactive oxygen species (ROS) [16,17,18,19]. Furthermore, airway exposure to β-glucan also causes tachypnea [5,13], implying the involvement of lung vagal sensory nerves. However, whether the β-glucan is acting on the lung vagal sensory nerves and then induces airway hypersensitivity remains unknown.

The capsaicin-sensitive lung vagal (CSLV) afferents innervating the lungs and airways are highly chemosensitive. They are important in detecting various insults to the lungs, such as ozone and cigarette smoke [20,21,22,23]. Stimulation of these afferents is known to elicit several reflex responses, such as bronchoconstriction and cough. Moreover, it has been reported that many endogenous mediators, such as ROS, are released during lung inflammation and known to produce sensitizing effects on CSLV afferents [24,25]. Once these afferents become more sensitive, several mediators released during lung inflammation will trigger airway hypersensitivity, which, characterized by a same level of stimulus, will elicit more intense airway reflexes [6,7,26,27]. Therefore, the sensitization of CSLV afferents is believed to contribute to airway hypersensitivity in airway inflammatory diseases, such as asthma [7,27].

The application of β-glucan has been demonstrated to induce allodynia, which was partially reduced in TRPA1-deficient mice, implying the involvement of the TRPA1 receptors [28]. Besides, we demonstrated that the activation of CSLV afferents induced by cigarette-smoke inhalation is mediated through the activation of TRPA1 receptors by ROS in our previous study [29]. However, whether TRPA1 and ROS contribute to the β-glucan-induced sensitization of CSLV afferents and consequent airway hypersensitivity remains unknown. To light up the information, this study is carried out to determine: (1) whether airway exposure to β-glucan by intratracheal instillation enhances the pulmonary chemoreflex responses in anesthetized, spontaneously breathing rats; if yes, (2) whether CSLV afferents play a role in the β-glucan-induced airway hypersensitivity (augmentation of pulmonary chemoreflex responses); (3) the role of ROS, TRPA1 receptors, and Dectin-1 receptors in the β-glucan-induced airway hypersensitivity; (4) whether β-glucan directly elevates the excitability of isolated CSLV neurons; (5) the Dectin-1 expression in the lung vagal neurons.

## 2. Results

### 2.1. In Vivo Study

The animals were divided into 13 groups to conduct 5 series of experiments. The in vivo study was carried out in 86 animals. Each group in study series 1–3 contained six animals; each group in study series 4–5 contained eight animals, and only one CSLV afferent fiber was studied in each animal. Table 1 shows various experimental interventions in the study groups tested.

**Figure 1:**
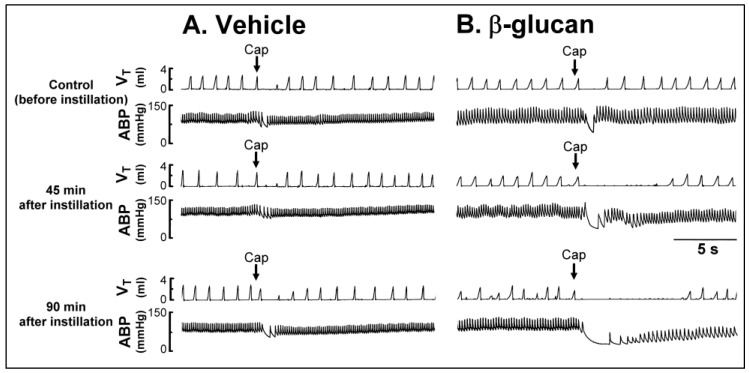
Experimental records illustrating the pulmonary chemoreflex responses to right-atrial injection of capsaicin (Cap, 1 μg/kg; arrows) before, 45 min after, and 90 min after intratracheal instillation of (**A**) vehicle (isotonic saline) or (**B**) β-glucan (5 mg/0.1 mL/rat) in two anesthetized, spontaneously breathing rats (vehicle: 305 g; β-glucan: 310 g). V_T_, tidal volume; ABP, arterial blood pressure. Please note that the pulmonary chemoreflex responses evoked by capsaicin were enhanced by the β-glucan instillation, but not altered by the vehicle instillation.

**Figure 2:**
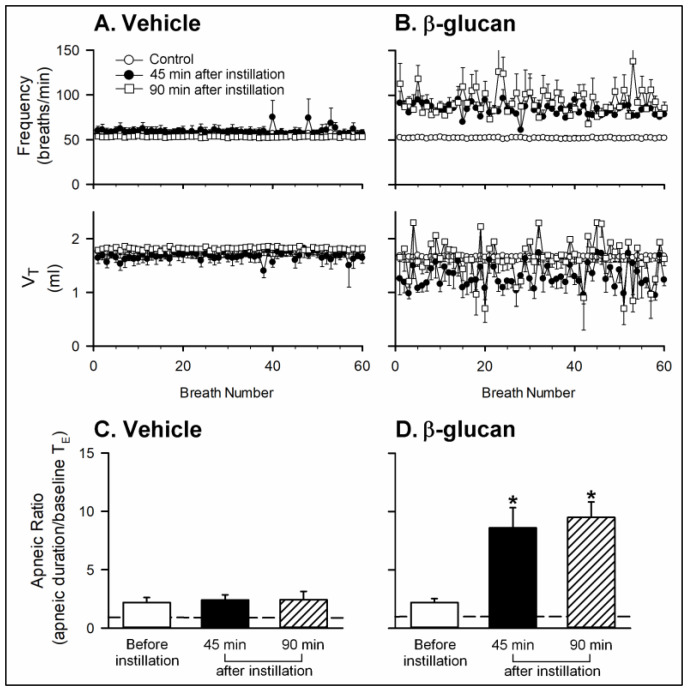
Effects of intratracheal instillation of β-glucan or its vehicle on the baseline breathing pattern and apneic response to right-atrial injection of capsaicin in two groups of anesthetized, spontaneously breathing rats. (**A**,**B**): effects of intratracheal instillation of vehicle or β-glucan on the baseline frequency and V_T_, respectively; (**C**,**D**): effects of intratracheal instillation of vehicle or β-glucan on the apneic responses to capsaicin injection, respectively. The apneic ratio was defined as the apneic duration occurring during 20 s after the capsaicin injections divided by the baseline expiratory duration (T_E_). Data are means ± SE (*n* = 6). One-way ANOVA followed by Newman-Keuls post hoc test: *, significantly different from before instillation (*p* < 0.05). Please note that the β-glucan instillation caused an irregular baseline breathing pattern, characterized by high respiratory frequency and unstable V_T_; β-glucan also enhanced the apneic responses.

Series 1. To investigate the enhancing effect of β-glucan on respiratory reflexes, the respiratory responses elicited by iv injection of capsaicin (a selective stimulant of CSLV afferents, 1 μg/kg) were compared before and after the airway instillation of β-glucan or its vehicle. In both vehicle and β-glucan groups, a right-atrial bolus injection of capsaicin triggered pulmonary chemoreflex response, characterized by a ventilatory inhibition (apnea, reduced respiratory frequency, reduced tidal volume) accompanied by transient hypotension and bradycardia (e.g., Figure 1). The apnea was displayed by several breaths with a prolongation of the expiratory duration (T_E_) (e.g., Figure 1). The apneic ratio was calculated by the apneic duration (the longest T_E_) occurring during the first 20 s after the capsaicin injections divided by the baseline T_E_ (apneic duration/baseline T_E_) (e.g., Figure 2). We found no significant difference in the baseline mean arterial blood pressure and heart rate (e.g., Figure 1; Table 2). However, the baseline breathing pattern became irregular 45 min after β-glucan instillation and lasted for 90 min, characterized by a high respiratory frequency and smaller, unstable tidal volume (Figure 2A,B). Forty-five minutes after β-glucan instillation (5 mg/0.1 mL), the capsaicin-elicited pulmonary chemoreflex responses were potentiated (Figure 1B and Figure 2B), and the potentiation sustained at least 90 min. The apneic ratio triggered by capsaicin significantly increased 45 min and 90 min after β-glucan instillation (Figure 2B,D, before: 2.20 ± 0.34; 45 min after: 8.60 ± 1.73; 90 min after: 9.50 ± 1.32; *n* = 6), but not altered by vehicle instillation (Figure 2A,C, before: 2.18 ± 0.44; 45 min after: 2.40 ± 0.45; 90 min after: 2.42 ± 0.71; *n* = 6).

**Figure 3:**
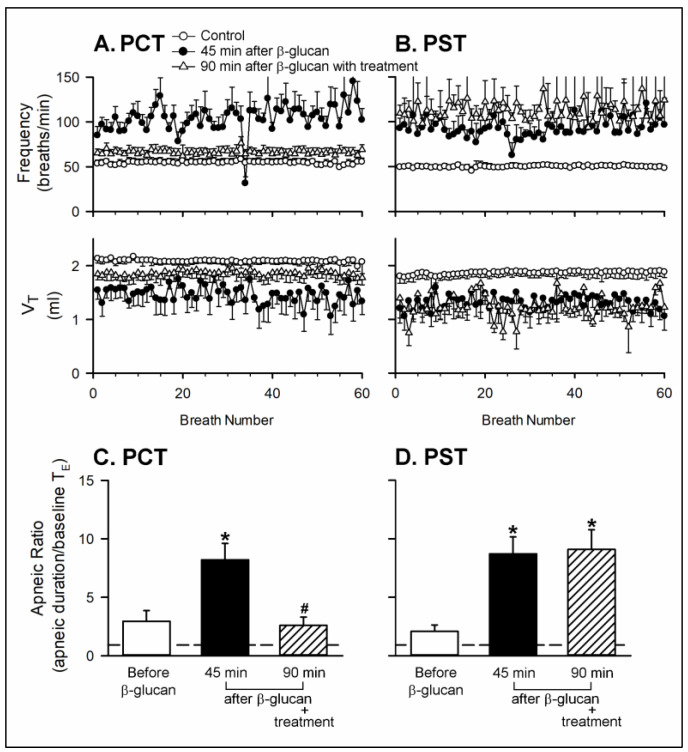
Effects of perineural capsaicin treatment (PCT) and perineural sham treatment (PST) on the enhancing effects of the intratracheal instillation of β-glucan on the baseline breathing pattern and apneic response to right-atrial injection of capsaicin in two groups of anesthetized, spontaneously breathing rats. (**A**,**B**): effects of PCT and PST on the intratracheal instillation of β-glucan on the baseline frequency and V_T_, respectively; (**C**,**D**): effects of PCT and PST on the intratracheal instillation of β-glucan on the apneic responses to capsaicin injection, respectively. Data are means ± SE (*n* = 6). One-way ANOVA followed by Newman-Keuls post hoc test: *, significantly different from before β-glucan (*p* < 0.05); ^#^, significant difference when corresponding data between without and with treatment were compared (*p* < 0.05). Please note that the β-glucan-induced sensitization was blocked by PCT, but not altered by PST.

Series 2. To investigate the role of CSLV afferents, the perineural capsaicin treatment (PCT) of the bilateral cervical vagi is a method modified from Jancso and Such, which can block the conduction of the CSLV afferents [8,30,31]. The irregular breathing pattern induced by β-glucan instillation was reversed by PCT (Figure 3A), but not altered by perineural sham treatment (PST) (Figure 3B). In addition, the PCT blocked β-glucan-induced enhancement of apnea evoked by capsaicin, and the PST failed to do so: the enhancing effect of β-glucan on the apneic ratio evoked by capsaicin was 8.20 ± 1.40 before PCT and 2.60 ± 0.71 after PCT (Figure 3C, *p* < 0.05; *n* = 6), respectively.

**Figure 4:**
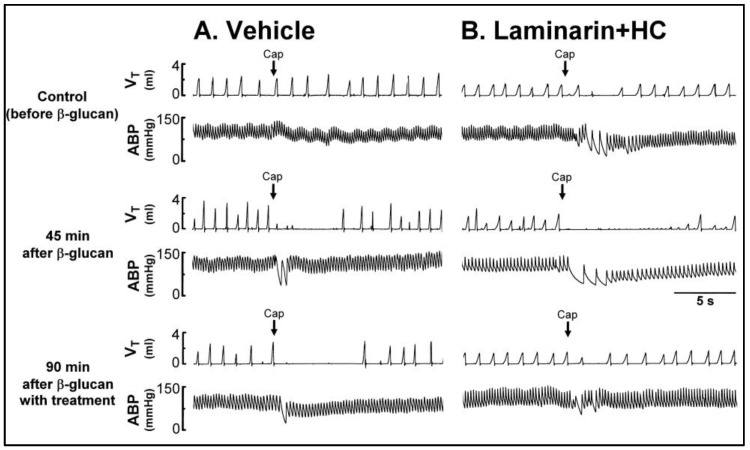
Experimental records illustrating the effect of treatment with (**A**) vehicle or (**B**) Laminarin (10 mg/kg, iv) + HC (8 mg/kg, iv) on the β-glucan-induced enhancement of pulmonary chemoreflex responses triggered by capsaicin injection (Cap, 1 μg/kg; arrows) in two anesthetized, spontaneously breathing rats (vehicle: 340 g; Laminarin + HC: 325 g). V_T_, tidal volume; ABP, arterial blood pressure. See the legend of Figure 1 for further explanation. Please note that the β-glucan-induced enhancement of pulmonary chemoreflex responses was abolished with Laminarin + HC treatment. HC, HC-030031.

**Figure 5:**
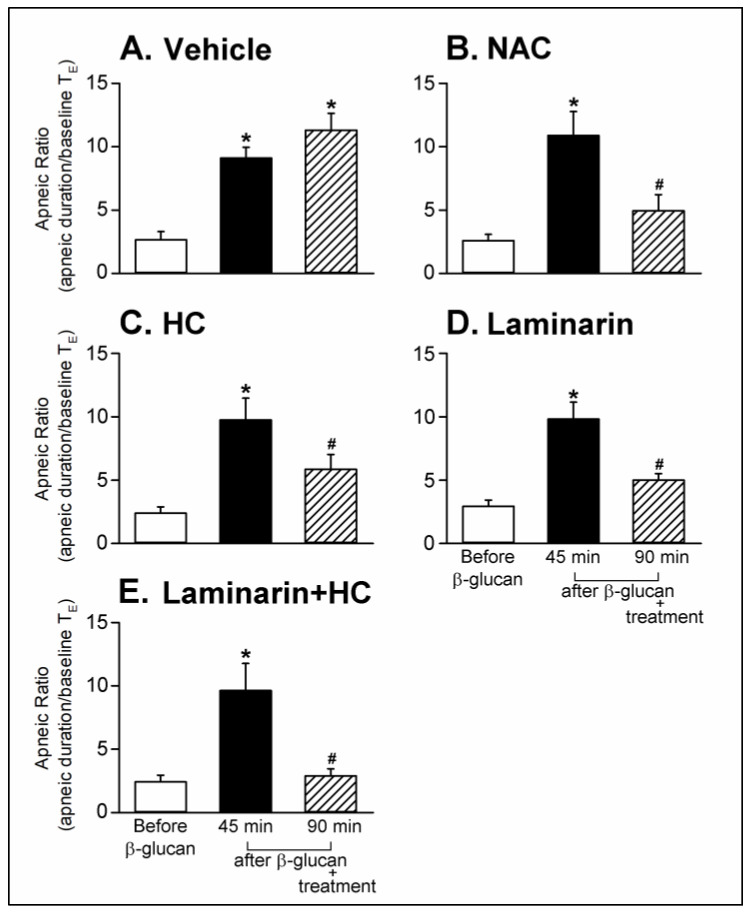
Effects of treatment with (**A**) vehicle, (**B**) NAC (0.3 g/kg, iv), (**C**) HC (8 mg/kg, iv), (**D**) Laminarin (10 mg/kg, iv) and (**E**) Laminarin + HC on the β-glucan-induced enhancement of pulmonary chemoreflex responses triggered by capsaicin injection in anesthetized, spontaneously breathing rats. Data are means ± SE (*n* = 6). One-way ANOVA followed by Newman-Keuls post hoc test: *, significantly different from before β-glucan (*p* < 0.05); ^#^, significant difference when corresponding data between without and with treatment were compared (*p* < 0.05). NAC, N-acetyl-L-cysteine; HC, HC-030031.

Series 3. To assess the roles of ROS, TRPA1 receptors, and Dectin-1 receptors in this enhanced airway response, the sensitizing effect of β-glucan on respiratory responses to capsaicin was investigated before and after treatment with N-acetyl-L-cysteine (NAC, a ROS scavenger), HC-030031 (a TRPA1 antagonist), Laminarin (a Dectin-1 antagonist), Laminarin + HC-030031, and the vehicle. The β-glucan-induced enhancement of pulmonary chemoreflex responses (e.g., top and middle panel of Figure 4) was nearly abolished by treatment with Laminarin + HC-030031 (Figure 4B and Figure 5E, the enhancing effect of β-glucan on the apneic ratio evoked by capsaicin before and after Laminarin + HC-030031 treatment: 9.63 ± 2.14 and 2.89 ± 0.56, *p* < 0.05; *n* = 6), whereas it was partially blocked by treatment with NAC, HC-030031, or Laminarin (Figure 5B–D). However, the vehicle did not affect the β-glucan-induced enhancement of pulmonary chemoreflex responses (Figure 4A and Figure 5A, the enhancing effect of β-glucan on the apneic ratio evoked by capsaicin before and after vehicle treatment: 9.10 ± 0.85 and 11.31 ± 1.32, *p* > 0.05; *n* = 6).

**Figure 6:**
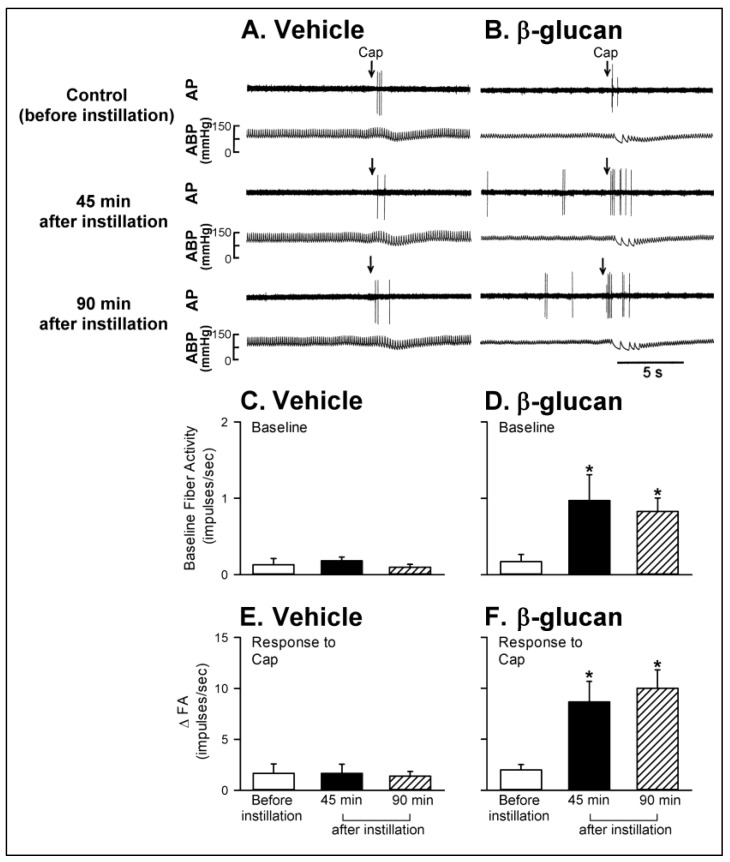
Experimental records illustrating the responses of capsaicin-sensitive lung vagal (CSLV) afferents to right-atrial injection of capsaicin (Cap, 0.75 μg/kg; arrows) before, 45 min after, and 90 min after intratracheal instillation of vehicle or β-glucan in anesthetized, artificially ventilated rats. (**A**,**B**): intratracheal instillation of vehicle or β-glucan (5 mg/0.1 mL/rat) in two anesthetized, artificially ventilated rats (vehicle: 350 g; β-glucan: 355 g), respectively. AP, action potential; ABP, arterial blood pressure. (**C**,**D**): the effects of vehicle and β-glucan on the baseline fiber activity of CSLV afferents, respectively. (**E**,**F**): the effects of vehicle and β-glucan on the responses of CSLV afferents to the capsaicin injection, respectively. FA, fiber activity; ∆FA, increase in the fiber activity was measured as the difference between peak FA (averaged over 3-s intervals) and the baseline FA (averaged over 10-s intervals) in each CSLV afferent. Data are means ± SE (*n* = 8). One-way ANOVA followed by Newman-Keuls post hoc test: *, significantly different from before instillation (*p* < 0.05). Please note that the β-glucan instillation caused an elevation in baseline FA; furthermore, the ∆FA triggered by capsaicin injection was also enhanced by β-glucan instillation.

Series 4. To investigate the enhancing effect of β-glucan on the CSLV afferents, the sensitizing effect of β-glucan on responses of CSLV afferents to capsaicin (0.75 μg/kg) were investigated before and after airway instillation of β-glucan or its vehicle. In both vehicle and β-glucan groups, a right-atrial bolus injection of capsaicin triggered a short and mild burst of discharge of CSLV afferents immediately (within 3 s) (e.g., upper panel of Figure 6). The baseline fiber activity (FA) of CSLV afferents was elevated after the airway instillation of β-glucan, since it evoked a stimulation of CSLV afferents (e.g., Figure 6B). In contrast, the instillation of the vehicle failed to cause this effect (e.g., Figure 6A). The responses of baseline FA of CSLV afferents before β-glucan, 45 min after β-glucan, and 90 min after β-glucan were 0.17 ± 0.09, 0.97 ± 0.34, and 0.83 ± 0.18 impulses/s (*p* < 0.05; *n* = 8), respectively. Forty-five minutes after β-glucan instillation into the lungs, the same dose of capsaicin evoked a significantly higher FA in the same CSLV afferent (Figure 6B); ∆FA triggered by the same dose of capsaicin was increased 4.33-fold (Figure 6F, from 2.00 ± 0.52 impulses/s to 8.67 ± 2.01 impulses/s, *p* < 0.05; *n* = 8). The β-glucan-induced augmentation in the FA of CSLV was sustained for 90 min (lower panel of Figure 6B); ∆FA triggered by the same dose of capsaicin was increased 5-fold (from 2.00 ± 0.52 impulses/s to 10.00 ± 1.81 impulses/s, *p* < 0.05; *n* = 8). In sharp contrast, the vehicle instillation, in the same manner, did not alter the FA responses of CSLV afferents to capsaicin at any time points following the same protocol (Figure 6A,E, *p* > 0.05; *n* = 8).

**Figure 7:**
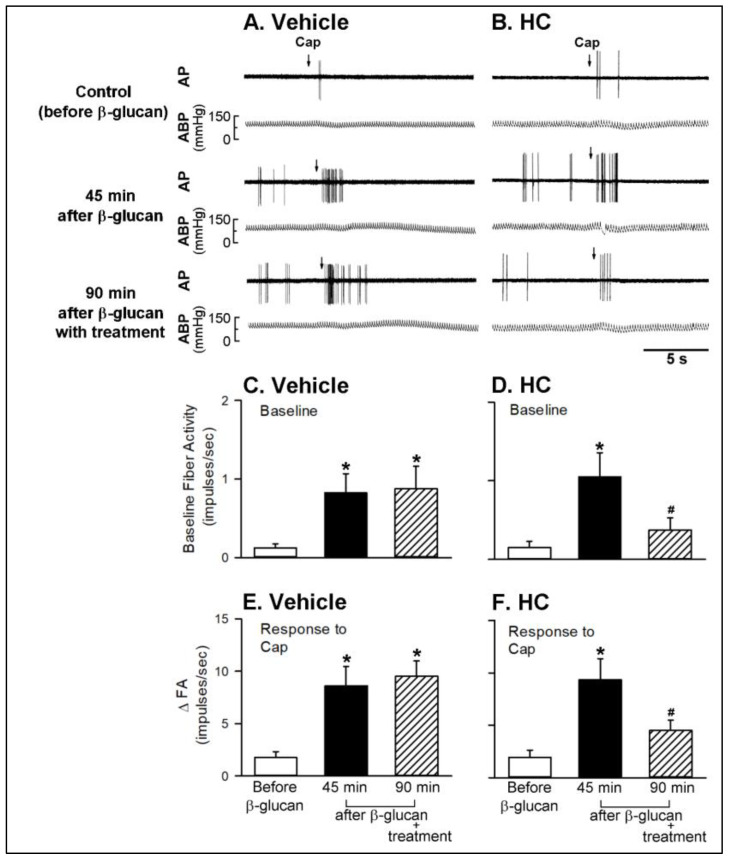
Experimental records illustrating the effects of treatment of vehicle or HC-030031 (HC, 8 mg/kg) on the β-glucan-induced potentiating on the responses of capsaicin-sensitive lung vagal (CSLV) afferents to capsaicin (Cap, 0.75 μg/kg; arrows) after intratracheal instillation of β-glucan (5 mg/0.1 mL/rat) in anesthetized, artificially ventilated rats. (**A**,**B**): treatment with vehicle or HC in two anesthetized, artificially ventilated rats (vehicle: 300 g; β-glucan: 290 g), respectively. AP, action potential; ABP, arterial blood pressure. (**C**,**D**): the effects of vehicle and HC on the β-glucan-induced elevation of the baseline fiber activity of CSLV afferents, respectively. (**E**,**F**): the effect of vehicle and HC on the β-glucan-induced potentiating on the responses of CSLV afferents to the capsaicin injection, respectively. FA, fiber activity. Data are means ± SE (*n* = 8). See the legend of Figure 6 for further explanation. One-way ANOVA followed by Newman-Keuls post hoc test: *, significantly different from before β-glucan (*p* < 0.05); ^#^, significant difference when corresponding data between without and with treatment were compared (*p* < 0.05). Please note that the HC treatment abolished the β-glucan-induced elevation of the baseline fiber activity of CSLV afferents; furthermore, the β-glucan-induced potentiating of the ∆FA of CSLV afferents triggered by capsaicin injection was also abrogated by HC treatment.

Series 5. The sensitizing effect of β-glucan on responses of CSLV afferents to capsaicin was investigated before and after treatment with HC-030031 and its vehicle to assess the roles of TRPA1 receptors in this enhancing effect. Treatment of HC-030031 significantly blocked the β-glucan-induced hypersensitivity of CSLV afferents (e.g., Figure 7); after the HC-030031 treatment, the effect of β-glucan on the ∆FA induced by capsaicin was 4.51 ± 0.99 impulses/s, which was significantly smaller than the ∆FA induced by capsaicin before HC-030031 treatment (9.33 ± 2.03 impulses/s, *p* < 0.05; *n* = 8). The vehicle of HC-030031 did not affect the β-glucan-induced hypersensitivity of CSLV afferents (Figure 7A,C,E).

### 2.2. In Vitro Study

The Ca^2+^ transients experiments were performed in 66 CSLV neurons (*n* = 22 in each group) isolated from the nodose and jugular ganglia of eight rats and identified by the fluorescent tracer of DiI. Because the Ca^2+^ transients’ responses were similar between nodose and jugular, the data were pooled together.

**Figure 8:**
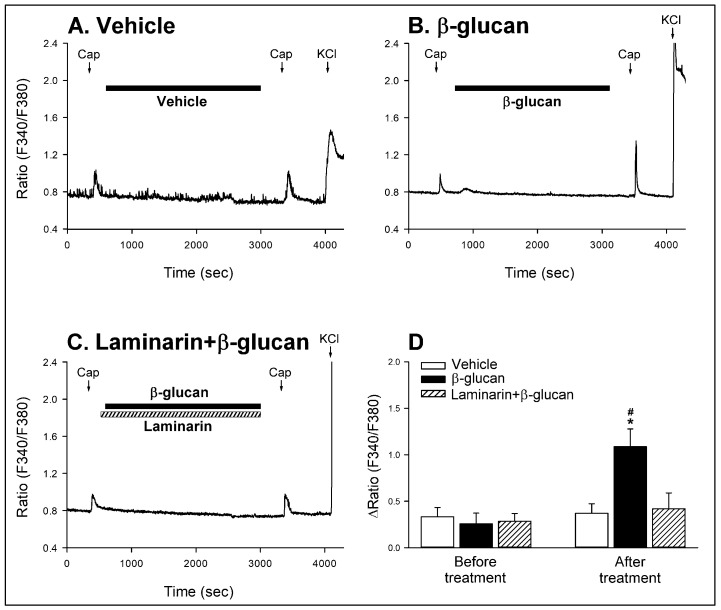
Experimental records illustrating the potentiating effect of β-glucan on capsaicin-evoked Ca^2+^ transients and the role of Dectin-1 receptors in this potentiating effect of β-glucan in isolated CSLV neurons. (**A**,**B**): perfusion with vehicle (ECS; filled horizontal bar) or β-glucan (200 μg/mL, 40 min; filled horizontal bar), respectively. Capsaicin (Cap, 0.1 μM, 30 s; arrows) was applied before and 5 min after the β-glucan or its vehicle perfusion, and a KCl solution (60 mM, 30 s; arrows) was applied to test the cell viability at the end of the experiment. (**C**): treatment with Laminarin (100 μg/mL, 41 min; hatched horizontal bar) + β-glucan; Cap was applied before and 5 min after Laminarin + β-glucan. (**D**): the role of Dectin-1 receptors in the potentiating effect of β-glucan on capsaicin-evoked Ca^2+^ transients in isolated CSLV neurons. An increase in 340/380 ratio [∆Ratio (F340/F380)] was measured as the difference between the peak amplitude of Ca^2+^ transients (4-s average) and the 30-s average at baseline. Data are means ± SE (*n* = 22). Two-way ANOVA followed by Newman-Keuls post hoc test: *, significantly different from vehicle (*p* < 0.05); ^#^, significant difference when corresponding data between before and after treatment were compared (*p* < 0.05).

Series 1. To examine the direct sensitizing effect of β-glucan on the CSLV neurons, Ca^2+^ transients elicited by capsaicin (0.1 μM, 30 s) were determined before and after the β-glucan perfusion. The application of capsaicin induced Ca^2+^ transient in CSLV neurons (e.g., Figure 8). Perfusion with the vehicle of β-glucan for 40 min did not alter the Ca^2+^ transient evoked by capsaicin (Figure 8A). As shown in Figure 8B, β-glucan application alone did not cause a detectable effect on the basal intracellular Ca^2+^ concentration. In contrast, after perfusion with β-glucan for 40 min, the Ca^2+^ transient evoked by capsaicin was significantly enhanced (Figure 8B,D); the responses of ∆ratio of Ca^2+^ transient were 0.26 ± 0.12 (F340/F380) and 1.09 ± 0.19 (F340/F380) before and after β-glucan perfusion, respectively (Figure 8D, *p* < 0.05; *n* = 22).

Series 2. To determine the role of Dectin-1 receptors, the β-glucan-induced potentiation of capsaicin-evoked Ca^2+^ transients were determined after the pretreatment of Laminarin. After pretreatment with Laminarin (Figure 8C,D), the β-glucan-induced hypersensitivity of CSLV neurons was largely reduced: the responses of ∆ratio of Ca^2+^ transient were 0.29 ± 0.08 (F340/F380) and 0.42 ± 0.17 (F340/F380) before and after Laminarin + β-glucan perfusion, respectively (Figure 8D, *p* > 0.05; *n* = 22). Moreover, the Laminarin alone did not influence the capsaicin-induced Ca^2+^ transient: the ∆ratios induced by capsaicin before and after Laminarin alone were 0.34 ± 0.12 and 0.36 ± 0.17 (F340/F380), respectively (*p* > 0.05; *n* = 11).

### 2.3. Immunofluorescence

**Figure 9:**
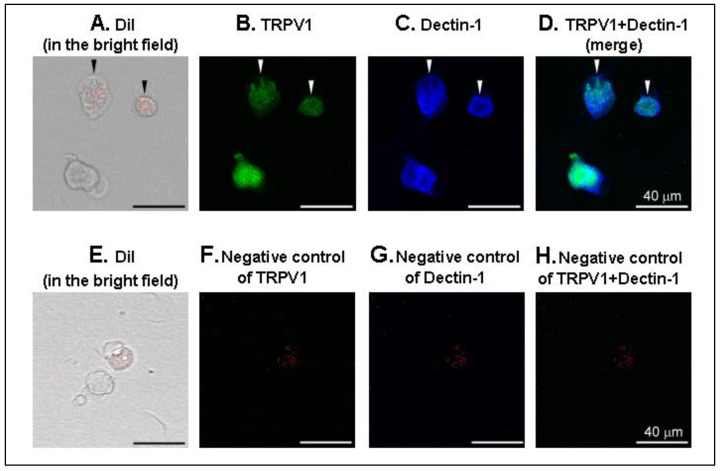
Representative photographs of triple-labeling immunofluorescence of DiI, TRPV1, and Dectin-1 in the lung vagal neurons. (**A**,**E**): DiI labeling in red in the bright field was served as positive control; (**B**) TRPV1 staining in green; (**C**) Dectin-1 staining in blue; (**D**) the merged image; (**F**–**H**): negative control of TRPV1, Dectin-1, and TRPV1 + Dectin-1, respectively. We used bovine serum albumin to demonstrate specific binding of primary antibody. Arrowheads are added to depict co-localization of DiI labeling, TRPV1 staining, and Dectin-1 staining in the same neurons. Scale bar, 40 μm.

We examined the Dectin-1 expression in isolated lung vagal neurons. The lung vagal neurons were identified using DiI labeling (Figure 9A). In isolated lung vagal neurons, 18.2% (59/324) of the neurons were retrogradely labeled with DiI. Then, 64.4% (38/59) presented TRPV1 immunoreactivity among these DiI-labeled neurons. We further observed co-localization of DiI labeling, TRPV1 staining, and Dectin-1 expression in the same neurons (Figure 9D).

## 3. Discussion

This study demonstrated that the intratracheal instillation of β-glucan potentiated the sensitivity of the CSLV afferents and in turn, enhanced the pulmonary chemoreflex responses. This sensitizing effect of β-glucan was significantly reduced by NAC, Laminarin, HC-030031, and further abolished by a combination of Laminarin and HC-030031, suggesting the involvement of the TRPA1 and the Dectin-1 receptors via an independent pathway. Furthermore, we observed the Dectin-1 expression in the isolated lung vagal neurons and perfusion of β-glucan enhanced the Ca^2+^ transients evoked by capsaicin challenge. Besides, consistent with our in vivo results, the pretreatment with Laminarin to antagonize the Dectin-1 receptors reversed the β-glucan-induced potentiation of Ca^2+^ transients in isolated CSLV neurons. In summary, these results suggest that β-glucan induces airway hypersensitivity through the activation of the TRPA1 receptors and Dectin-1 receptors. Moreover, a direct action on the Dectin-1 receptors expressed in the CSLV afferents, at least in part, mediated the β-glucan-induced sensitization.

It has been shown that β-glucan induced lung damage (e.g., damage in alveolar-capillary barrier), lung inflammation (e.g., infiltration of polymorphonuclear leukocytes in lungs), accompanied by tachypnea after 18 or 24 h of intratracheal instillation [5,13], implying the involvement of lung vagal sensory nerves in this β-glucan-induced responses [5,13]. However, there was no evidence of the β-glucan-induced effect on these sensory nerves. Here we showed the first evidence that β-glucan applied by intratracheal instillation exhibited sensitizing effects on the CSLV afferents, and these sensitizing effects were mediated through the TRPA1 and Dectin-1 receptors. In the Ca^2+^ transients’ experiment, we demonstrated that β-glucan exerted a sensitizing effect directly on the isolated CSLV neurons. We further discovered Dectin-1 receptors expression in isolated lung vagal TRPV1-positive neurons, implying that β-glucan might act directly on the CSLV neurons mediated through these Dectin-1 receptors. Indeed, the β-glucan-induced sensitizing effect could be inhibited by Laminarin, a Dectin-1 antagonist. Dectin-1 is expressed on monocytes, neutrophils of the blood, bone marrow, spleen, and dendritic cells [32,33]. Dectin-1 is also expressed abundantly on the alveolar and inflammatory macrophage, which may imply the involvement of Dectin-1 in the immune surveillance [12]. More importantly, Dectin-1 was demonstrated to mediate cellular responses to β-glucan, which acts as a potent pro-inflammatory inducer and immunoregulator to induce the production of various inflammatory cytokines released from leukocytes and other tissues, including ROS [18,34,35,36], TNFα [17,18,19], NF-κB [18], IL-8 [17,19], and IL-12 [19]. Therefore, the activation of cytokine mechanism and immunoregulatory intracellular signaling pathway was believed to be responsible for the β-glucan-caused Dectin-1-mediated adverse pulmonary responses over the past decade [10,11,18].

More recently, the expression of Dectin-1 has been shown in isolated dorsal root ganglion (DRG) neurons [28,37]. However, there is no report of Dectin-1 expression in the lung vagal sensory nerve endings, which believably play a critical role in the defensive reflex response to fungal exposure. Indeed, the lung vagal sensory nerves may directly sense fungal invasion like innate immune cells earlier if there is Dectin-1 expression in the lung vagal sensory nerves. In fact, in the present study, the β-glucan-enhanced sensitivity of CSLV afferents and pulmonary chemoreflexes was relatively speedy—it was found at the 45 min after application and persisted at 90 min. More importantly, the β-glucan-induced direct sensitizing effect on Ca^2+^ transients in the CSLV neurons occurred 5 min after 40-min-β-glucan perfusion. We also observed the Dectin-1 immunoreactivity on the TRPV1-positive lung vagal neurons, which highly supported the role of neuronal Dectin-1, at least in part, in this β-glucan-induced direct sensitizing effect on the CSLV neurons. These findings here suggest that airway exposure to β-glucan may participate in a direct sensitizing effect on the CSLV afferents via the neuronal Dectin-1 receptors and in turn trigger airway hypersensitivity. This notion is supported by other studies that have shown that β-glucan application directly activates nociceptors in DRG cultured neurons in a Dectin-1 manner [37]. Besides this, β-glucan activates the DRG neurons and leads to calcitonin gene-related peptide (CGRP) production. However, it is still not fully understood how Dectin-1 mediates these effects in the CSLV afferents of β-glucan.

In this present study, we could not rule out the possibility that β-glucan-caused sensitization resulted from the indirect effects of inflammatory cytokines. The β-glucan also induces allodynia, which is the consequence of the sensitization of DRG neurons [28], and the allodynia induced by β-glucan can be abolished in Dectin-1 deficient animals. Other investigators demonstrated that β-glucan recognition by Dectin-1 triggered ROS production from leukocytes [34,35,36]. ROS is a critical inflammatory mediator involved in various lung inflammatory responses [29,38]. In fact, in this study, the β-glucan-induced enhancement of apneic responses triggered by capsaicin was inhibited by NAC, a ROS scavenger, suggesting that the β-glucan-induced airway hypersensitivity is mediated through the production of ROS. Moreover, the TRPA1 receptor is a Ca^2+^-permeable, nonselective transmembrane cation channel [39,40]. Originally, TRPA1 is predominantly expressed in the TRPV1-positive lung vagal neurons [39], and has been suggested to act as an oxidant sensor allowing the detection of lung ROS [24,29,40]. Therefore, in the in vivo study, the activation of Dectin-1 by β-glucan instillation may trigger the release of intermediate mediator(s) such as ROS from other target cells in the airways, which could in turn sensitize CSLV afferents via activation of TRPA1 and consequently lead to airway hypersensitivity. In addition, it is well documented that TRPA1 is also expressed in the non-neuronal cells, such as lung epithelial cells, lymphocytes, etc. [41,42]. Thus, we cannot exclude other possibilities that the activation of non-neuronal TRPA1 receptors may elicit the release of inflammatory mediators, which may then lead to the sensitization of CSLV afferents. Furthermore, in mouse DRG neurons, β-glucan was reported to evoke releases of pro-inflammatory neuropeptides, such as CGRP [37]. Hence, a possibility that the β-glucan-induced sensitization is because of its indirect action on the released inflammatory mediator(s) should be considered. Besides, in the present study, via activation of TRPA1, β-glucan-related ROS elevated the CSLV-neuron responses to TRPV1 agonist. Fischer and coworkers have reported that TRPA1 exerts a functional inhibition of TRPV1 by forming TRPA1/TRPV1 heteromers [43]. In our literature review, β-glucan or ROS did not down-regulate TRPA1 expression or modulate the heteromer formation. Thus, whether this heteromer plays a role in the β-glucan-induced sensitization of CSLV neurons needs further investigation.

As compared to the latency of β-glucan-induced hypersensitive response in isolated CSLV neurons, the latency in anesthetized rats is much longer (>45 min). The mechanism(s) involved in the long latency response of intratracheal instillation of β-glucan is still unknown, which may be associated with airway epithelial barrier [44,45]. The barrier-protective function is disrupted in several pathological conditions, such as asthmatic patients [45,46] and cigarette smokers [47]. This may strengthen the importance of CSLV neurons in detecting and triggering early airway responses to fungal insults.

It is believed that the risk of opportunistic fungal infection in the lungs is relatively less because the immune system is equipped to fight and clear living fungi [48]. However, lung exposure to fungal allergens is a common cause of airway hypersensitive diseases, such as asthma [49,50], which exaggerate response to numerous stimuli to the lung. The most attractive fungal antigen is 1,3-β-D-glucan, in which concentration was highly associated with the severity of airway hypersensitive diseases [51,52]. Our results showed that Dectin-1 antagonist largely suppressed hypersensitivity of airways and CSLV afferents/neurons after β-glucan exposure, suggesting a therapeutic potential of the Dectin-1 antagonist. However, Dectin-1 plays a vital role in the innate immunity of antifungal infection [53]. Thus, over-suppression of Dectin-1 may result in a liability to fungal infection in the lungs. In fact, the genetic intervention of Dectin-1 receptors expands the pathogenic commensal fungi in the gut [54]. Therefore, for balance between the benefits and the risks for medication use, the Dectin-1 antagonists should be used with caution in patients with fungal-related airway hypersensitivity. Accordingly, the TRPA1 antagonists will allow the future exploitation of the therapeutic potential for the fungal-related airway hypersensitive diseases.

## 4. Materials and Methods

The following procedures were performed following the recommendations found in the “Guide for the Care and Use of Laboratory Animals” published by the National Institutes of Health and were approved by the Institutional Animal Care and Use Committee of Taipei Medical University (2015-0411).

### 4.1. In Vivo Study

#### 4.1.1. General Preparation

Male Sprague-Dawley rats (300–350 g) were initially anesthetized with an ip injection of α-chloralose (100 mg/kg) and urethane (500 mg/kg). A short cannula was inserted via a tracheostomy. The right femoral artery was cannulated for measuring arterial blood pressure. The right jugular vein was cannulated for the administration of anesthetics and pharmacological agents. Body temperature was maintained at ~36 °C throughout the experiments using a heating pad placed under the rat lying in a supine position.

#### 4.1.2. Airway Exposure to β-glucan

Solution of β-glucan was instilled into the trachea (5 mg/rat, in a volume of 0.1 mL; Sigma) by using the Hamilton Microsyringe (Hamilton^®^, Reno City, NV, USA). The control animals were instilled with the sterile isotonic saline (the vehicle of β-glucan).

#### 4.1.3. Measurement of Ventilatory Responses

Animals breathed spontaneously via the tracheal cannula. Ventilatory flow was measured with a heated pneumotachgraph and a differential pressure transducer and was integrated to given tidal volume (V_T_). Respiratory frequency, V_T_, heart rate, and arterial blood pressure were analyzed on a breath-by-breath basis. These ventilatory and cardiovascular signals were recorded on a polygraph recorder (MP30; BIOPAC, Instrument, Goleta, CA, USA) and also analyzed by an on-line computer (TS-100; BioCybernetics, Taipei, Taiwan). Before each measurement, the lungs were hyperinflated (tracheal pressure > 10 cmH_2_O) to establish a constant volume history. For the ventilatory responses’ comparison, the T_E_ was analyzed on a breath-by-breath basis over an interval of at least 20 breaths before and 60 breaths after chemical injection. The baseline T_E_ was calculated by the average value over the 10-breath duration before the chemical injection. To compare the apneic response elicited by different experimental conditions, the longest T_E_ occurring during the first 20 s after chemical injection was divided by the baseline T_E_ to produce the apneic ratio.

#### 4.1.4. Perineural Capsaicin Treatment (PCT) of Cervical Vagi

To assess the role of CSLV afferents, the PCT was used to provide a differential blockade of the neural conduction on CSLV of bilateral cervical vagi [8,30,31]. Briefly, cotton strips soaked in a high concentration of capsaicin solution (250 μg/mL) or its vehicle (perineural sham treatment, PST) were wrapped around a 2- or 3-mm segment of the isolated cervical vagi. The criterion for a successful capsaicin treatment was judged on the abolition of the reflex responses induced by the right-atrial injection of capsaicin (a specific stimulant of CSLV afferents) and the existence of the Hering-Breuer reflex [55,56].

#### 4.1.5. Measurement of Excitability of CSLV Afferents

The excitability (fiber activity) of CSLV afferents was recorded in anesthetized, artificially ventilated rats by using the single-fiber recording technique described in our previous study [29,57]. Briefly, the trachea was cannulated and the lungs were artificially ventilated by a respirator after a tracheotomy. Tidal volume and respiratory frequency were set at 8 mL/kg and 50 breaths/min, respectively. Tracheal pressure was recorded via the sidearm of the tracheal cannula. The chest was opened to identify the location of the CSLV afferents and the expiratory outlet of the respirator was placed under 3-cmH_2_O pressure to maintain a near-normal functional residual capacity. The right cervical vagus nerve was separated from the carotid artery, sectioned rostrally, and placed in a small dissecting platform. With the aid of a dissecting microscope (SV6, Carl Zeiss, Germany) and fine-tip forceps, a thin filament was teased away from the desheathed nerve trunk and placed on a platinum-iridium hook electrode. Signals of the action potential were amplified, monitored by an audio monitor and displayed on an oscilloscope. The thin filament was further split until the fiber activity arising from a single unit was electrically isolated. Because the baseline discharge of CSLV afferents was usually sparse and irregular, hyperinflation of the lung (3–4 V_T_) was used as the first step to search for these fibers; the CSLV afferents were activated by lung inflation at this high-volume level [27]. Once the fiber activity of a single unit was identified by hyperinflation, capsaicin (a specific stimulant of CSLV afferents; 1.5 μg/kg) was injected intravenously into the right atrium via the jugular vein. Only fibers that met the following criteria were being studied in this study: (1) fibers with a short latency and intense response to capsaicin injection, and (2) at the end of the experiment, the general locations of the fibers could be identified by their responses to a gentle pressing of the lungs with a wet cotton swab. These criteria were established in our previous studies for the reliable identification of CSLV afferents [29,57]. The fiber activity (FA) of CSLV afferents was continually analyzed at 1-s intervals over an interval of 20 s before and 60 s after chemical injection. Baseline FA was calculated by the average value over the 10-s duration before the chemical injection. The peak response was defined as the maximum 3-s average response during 20-s following the chemical injection.

#### 4.1.6. Experimental Protocols

The β-glucan (5 mg/0.1 mL) was instilled into the lungs via Hamilton Microsyringe. Table 1 shows various experimental interventions in the study groups tested (Table 1). Series 1: to investigate the enhancing effect of β-glucan on respiratory reflexes, the respiratory responses elicited by iv bolus injection of capsaicin (a selective stimulant of CSLV afferents, 1 μg/kg) were compared 15 min before, 45 min after, and 90 min after the termination of airway instillation of β-glucan or its vehicle (isotonic saline) (group 1–2). Series 2: to investigate the role of CSLV afferents, the β-glucan-induced sensitizing effect on respiratory responses to capsaicin were compared before and after PCT (group 3) or PST (group 4). Series 3: the sensitizing effect of β-glucan on respiratory responses to capsaicin were investigated before and after treatment with N-acetyl-L-cysteine (NAC, 0.3 g/kg, iv), HC-030031 (8 mg/kg, iv), Laminarin (10 mg/kg, iv), Laminarin + HC-030031, and the vehicle to assess the roles of ROS, TRPA1 receptors, and Dectin-1 receptors in this enhanced airway responses (group 5–9). Series 4: the sensitizing effect of β-glucan on responses of CSLV afferents to capsaicin were investigated 15 min before, 45 min after, and 90 min after the termination of airway instillation of β-glucan or its vehicle (group 10–11). Series 5: the sensitizing effect of β-glucan on responses of CSLV afferents to capsaicin were investigated before and after treatment with HC-030031 and its vehicle to assess the roles of TRPA1 receptors in this enhancing effect (group 12–13).

### 4.2. In Vitro Study

The following experiments were performed in vitro preparation to assess whether β-glucan exerts a sensitizing effect directly on the CSLV neurons, and to determine the Dectin-1 expression on the CSLV neurons.

#### 4.2.1. Identification of CSLV Neurons

Sensory neurons innervating the airways and lungs were identified by retrograde labeling from the lungs with the fluorescent tracer 1,1′-dioctadecyl-3,3,3′,3′-tetramethylindocarbocyanine perchlorate (DiI). Young male SD rats (50~120 g) were anesthetized by aerosolized isoflurane (2% in O_2_) through a nosecone connected to a vaporizing machine. To expose the trachea, a small midline incision was made on the neck skin. The DiI (0.2 mg/mL; 0.05 mL; 1% ethanol concentration) was instilled into the lungs through a needle inserted into the trachea lumen, and the incision was then closed. To allow the DiI to be transported toward the soma of the CSLV neurons, the animals were kept undisturbed for 7–10 days until they were euthanized for the cell culture [58,59,60].

#### 4.2.2. Isolation and Culture of Nodose and Jugular Ganglion Neurons

The methodology of isolation and culture of nodose and jugular ganglion neurons was described in detail in our previous studies [58,59,60]. At 7–11 days, the DiI-labeled SD rats were anesthetized with 5% isoflurane and decapitated. The head was immediately immersed in ice-cold DMEM/F-12 solution. Nodose and jugular ganglia were desheathed, cut, placed in a mixture of collagenase (0.04%) and dispase II (0.02%), and incubated in 5% CO_2_ in air at 37 °C. The ganglion suspension was centrifuged (150 g, 5 min) and the supernatant aspirated. The cell pellet was then resuspended in a modified DMEM/F-12 solution and gently triturated. The dispersed cell suspension was centrifuged (500 g, 8 min) through a layer of 15% bovine serum albumin to separate the cells from the myelin debris. The pellets were resuspended in the modified DMEM/F-12 solution plated onto poly-L-lysine-coated glass coverslips and then incubated overnight in 5% CO_2_ in air at 37 °C.

#### 4.2.3. Measurement of Ca^2+^ Transients on Isolated CSLV Neurons

The measurement of Ca^2+^ transients on isolated CSLV neurons was described in detail in our previous studies [60]. Briefly, Ca^2+^ transients were measured in CSLV neurons with a Leica digital fluorescence microscope (DM IL LED; Leica Microsystems, Wetzlar, Germany) and a digital CCD camera (Zyla 4.2 sCMOS; Andor Technology, Belfast, UK). Neurons were washed or maintained with an extracellular solution (ECS) in a small-volume (0.2 mL) perfusion chamber at room temperature. Neurons were loaded with 5 μM fura-2 AM (Molecular Probes; Eugene, OR, USA) for 30 min at 37 °C in tissue culture medium and then rinsed with ECS and allowed to deesterify for at least 30 min before use. The recording chamber was perfused continuously with ECS or the test chemicals by a gravity-fed valve control system (VC-66CS; Warner Instruments, Hamden, CT, USA). Dual images (340-and 380-nm excitation, 510-nm emission) were collected, and pseudocolored ratiometric images were monitored during the experiments. The image signals were continually analyzed at 2-s intervals during the experiments by using the MetaFluor^®^ software (Universal imaging; West Chester, PA, USA). An increase in 340/380 ratio [∆Ratio (F340/F380)] was measured as the difference between the peak amplitude of Ca^2+^ transients (4-s average) and the 30-s average at baseline.

#### 4.2.4. Experimental Protocols

The neurons for analysis in this study were selected from the cultured cells that met the following criteria: (1) a spherical shape with no neurite outgrowths, (2) labeled with DiI fluorescence, and (3) activated by capsaicin (0.1 μM, 30 s). A total of 66 CSLV neurons were studied in four separate series of experiments. Series 1: to examine the sensitizing effect of β-glucan on the CSLV neurons, Ca^2+^ transients elicited by capsaicin (0.1 μM, 30 s) were determined before and 5 min after the β-glucan perfusion (200 μg/mL, 40 min) (group 1–2). Series 2: to determine the role of Dectin-1 receptors, the β-glucan-induced potentiation of capsaicin-evoked Ca^2+^ transients were determined after the pretreatment of Laminarin (100 μg/mL, 41 min) (group 3). The Laminarin was applied 1 min before and during β-glucan perfusion.

### 4.3. Immunofluorescence

Isolated vagal neurons from DiI-labeled SD rats were first fixed with 4% paraformaldehyde in phosphate-buffered saline (PBS) for 10 min. After PBS washing, neurons were permeabilized in 0.1% Triton X-100 for 10 min, washed with PBS, and blocked with PBS containing 5% bovine serum albumin (BSA) for 1 h at room temperature. Next, the neurons were incubated with a mixture of rabbit anti-Dectin-1 antibody (1:50; Abcam, Cambridge, UK) and mouse anti-TRPV1 antibody (1:100; Abcam) overnight at 4 °C, which were diluted in PBS containing 5% BSA. After three washes with PBS, the neurons were incubated with a mixture of Alexa Fluor^®^ 405-conjugated goat anti-rabbit IgG (1:200; Abcam) and Alexa Fluor^®^ 488-conjugated goat anti-mouse IgG (1:200; Abcam) for 1 h at room temperature. The neurons were rewashed by PBS and mounted in Aqueous Mounting Medium (Abcam). Images were captured using a confocal spectral microscope (TCS SP5; Leica Microsystems, Mannheim, Germany) with a 20× objective.

### 4.4. Pharmacological Agents

For the in vivo study, stock solutions of chemical agents were prepared as follows: capsaicin (250 μg/mL; Sigma) was prepared in 1% Tween 80, 1% ethanol, and 98% saline; NAC (0.15 g/mL; Sigma) was dissolved in saline; HC-030031 (30 mg/mL; Tocris) was dissolved in DMSO; Laminarin (10 mg/mL; Sigma) was dissolved in saline. Solutions of these pharmacological agents at the desired concentrations were prepared daily by dilution with saline based on the animal’s body weight, except the HC-030031 was further diluted to a final concentration of 2 mg/mL with a vehicle (10% Tween 80, 10% ethanol, and 80% saline) before use. For the in vitro study, desired concentrations of the pharmacological agents were prepared similarly, except that ECS, instead of saline, was used as the vehicle.

### 4.5. Data Analysis

The data were analyzed with t-test, one-way repeated measures ANOVA, or two-way ANOVA, followed by a post hoc Newman-Keuls test, unless mentioned otherwise. A value of *p* < 0.05 was considered to be significant. All data are means ± SE.

## 5. Conclusions

Our findings provide novel information that will allow a better understanding of the pathogenic mechanisms associated with β-glucan-induced airway hypersensitive diseases and should help the development of potential therapies.

## Figures and Tables

**Table 1:** Summary of the various experimental interventions in the study groups.

Study	Response	Series	Group	Instillation (In Vivo)/Perfusion (In Vitro)	Stimulus	Treatment
In vivo	Pulmonary chemoreflex	1	1	β-glucan	Capsaicin	-
			2	Vehicle of β-glucan	Capsaicin	-
		2	3	β-glucan	Capsaicin	PCT
			4	β-glucan	Capsaicin	PST
		3	5	β-glucan	Capsaicin	Vehicle
			6	β-glucan	Capsaicin	NAC
			7	β-glucan	Capsaicin	HC
			8	β-glucan	Capsaicin	Laminarin
			9	β-glucan	Capsaicin	Laminarin + HC
	Fiber activity	4	10	β-glucan	Capsaicin	-
			11	Vehicle of β-glucan	Capsaicin	-
		5	12	β-glucan	Capsaicin	Vehicle of HC
			13	β-glucan	Capsaicin	HC
In vitro	Ca^2+^ transient	1	1	β-glucan	Capsaicin	-
			2	Vehicle of β-glucan	Capsaicin	-
		2	3	β-glucan	Capsaicin	Laminarin

PCT, perineural capsaicin treatment; PST, perineural sham treatment; NAC, N-acetyl-L-cysteine; HC, HC-030031.

**Table 2:** Effects of intratracheal instillation of β-glucan and its vehicle on the baseline of mean arterial blood pressure (MABP) and heart rate (HR) in anesthetized, spontaneously breathing rats.

	Vehicle of β-Glucan (*n* = 6)	β-Glucan (*n* = 48)
**MABP, mmHg**		
Before instillation	117 ± 5	114 ± 2
45 min after instillation	113 ± 6	107 ± 2
90 min after instillation	114 ± 4	106 ± 2
**HR, beats/min**		
Before instillation	348 ± 23	365 ± 4
45 min after instillation	354 ± 30	356 ± 6
90 min after instillation	347 ± 33	353 ± 7

Data (means ± SE) are values averaged over 10-s periods before and 45 min and 90 min after the termination of instillation. *n* represents rat numbers. No statistical significance was found between any two groups (*p* > 0.05 with *t*-test).

## Abbreviations

CSLV	capsaicin-sensitive lung vagal
ROS	reactive oxygen species
TRPA1	transient receptor potential ankyrin 1
iv	intravenous
TRPV1	transient receptor potential vanilloid 1
ip	intraperitonal
DRG	dorsal root ganglion
CGRP	calcitonin gene-related peptide
